# Microencapsulation and characterization of pomegranate seed oil using gum Arabic and maltodextrin blends for functional food applications

**DOI:** 10.1002/fsn3.4493

**Published:** 2024-09-30

**Authors:** Kutloano Mangope, Tafadzwa Kaseke, Olaniyi A. Fawole

**Affiliations:** ^1^ South African Research Chairs Initiative in Sustainable Preservation and Agroprocessing Research, Faculty of Science University of Johannesburg Johannesburg South Africa; ^2^ Postharvest and Agroprocessing Research Centre, Department of Botany and Plant Biotechnology University of Johannesburg Johannesburg South Africa

**Keywords:** fatty acid composition, ferric reducing antioxidant power, functional foods, hygroscopicity, pomegranate seed oil encapsulation, total phenolic content

## Abstract

Pomegranate seed oil (PSO) is highly valued in the functional food industry due to its rich fatty acid content and associated health benefits. However, its high degree of unsaturation makes it susceptible to rapid degradation when exposed to oxygen and light. This study investigates the encapsulation of PSO at 15% w/w using different blends of gum Arabic (GA) and maltodextrin (MD) (1:0, 0:1, 1:1, 3:1, and 1:3) to determine optimal formulations for enhanced stability and functional quality. Characterization of the encapsulated PSO powders showed distinct particle morphologies, including flake‐like shapes and textures ranging from smooth to wrinkled and porous. The Fourier transform infrared (FT‐IR) spectra indicated shifts in functional groups from 2973.70 to 408.84 cm^−1^, revealing the presence of aliphatic, amine, aromatic, carboxylic acid, and hydroxyl groups. Although no single formulation achieved all desired outcomes, the GA:MD ratios of 1:0 and 1:1 were superior in enhancing color properties (yellowness and chroma), techno‐functional attributes (bulk density and solubility), and in preserving essential fatty acids, including stearic, cis‐oleic, α‐linolenic, arachidic, γ‐linolenic, linoleic, and punicic acids. Additionally, GA:MD (3:1) powders exhibited superior ferric‐reducing antioxidant power (FRAP) and 2,2‐diphenyl‐1‐picrylhydrazyl (DPPH) radical scavenging activities (RSA). In conclusion, formulations using solely GA or GA:MD ratios of 1:1 and 3:1 effectively preserve bioactive content in PSO, enhancing its antioxidant capacity. These findings suggest promising applications for these encapsulated powders in developing functional foods that meet industry demands.

## Graphical abstract

This study explores the encapsulation of pomegranate seed oil (PSO) using various blends of gum Arabic (GA) and maltodextrin (MD) to enhance its stability and functional quality. Formulations with GA ratios of 1:0, 1:1, and 3:1 were most effective in preserving essential fatty acids, improving color and techno‐functional properties, and boosting antioxidant capacity. These encapsulated powders hold the potential for developing functional foods that leverage the health benefits of PSO.

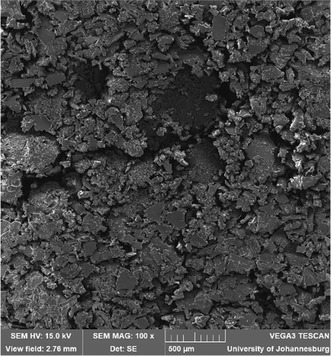

## 1 INTRODUCTION

Pomegranates (*Punica granatum* L.) have been valued since ancient Mediterranean civilizations for their delicacy and significant health benefits (Kandylis & Kokkinomagoulos, 2020). Processing pomegranate fruit into value‐added products like jam, jelly, juice, syrup, vinegar, and wine generates substantial by‐products, such as seeds and peels, typically considered waste.

Pomegranate seeds contain 12%–24% edible oil, rich in phytosterols, tocopherols, polyphenols, and punicic acid (Arendse et al., 2021; Boroushaki et al., 2016). Unique to pomegranate and gourd seed oils, punicic acid (C18:3, c9, t11, c13) is an isomer of conjugated linolenic acid (CLnA) and an omega‐5 long‐chain polyunsaturated fatty acid, structurally related to conjugated linoleic acid (CLA) and linolenic acid (LnA) (Viladomiu et al., 2013). Research indicates that punicic acid possesses antioxidant, anti‐diabetic, lipid metabolism‐regulating properties, estrogenic content, immune function, lipoperoxidation, and skin photoaging inhibition (Koba & Yanagita, 2011). Despite these benefits, punicic acid is prone to oxidation when exposed to oxygen, light, high temperatures, and moisture (Cortez‐Trejo et al., 2021). Encapsulation is essential to mitigate oxidation and preserve the valuable compounds in PSO during storage. Encapsulation has been used to protect sensitive plant material components from harsh environmental conditions (Premi & Sharma, 2017).

Various encapsulants, including polysaccharides, lipids, and proteins, have been utilized to improve the stability of seed oils for industrial applications. Common encapsulation methods for seed oils include spray drying and freeze drying (Rout et al., 2021). Freeze drying, the method chosen for this study, is preferred for its minimal heat usage, crucial for heat‐sensitive products like volatiles and oils (Fang & Bhandari, 2012). Selecting suitable wall materials and encapsulation techniques is vital to successfully incorporating bioactive phytochemicals into capsules (Buljeta et al., 2022). Polysaccharides such as GA and MD are widely used due to their diversity, low cost, and excellent properties for encapsulating bioactive compounds from plant materials.

GA is favored as a hydrocolloid‐based coating material due to its high solubility in water, low viscosity, and good emulsifying properties (Cano‐Chauca et al., 2005). Conversely, MD provides good protection against oxidation and maintains low viscosity at high concentrations (Kang et al., 2019), but its low emulsification properties necessitate blending with other materials like GA to produce stable emulsions. Blending polymers is increasingly becoming a preferred method of encapsulating plant extracts as it produces wall materials with customized properties, improved functionality, and specific advantages by exploiting the unique characteristics of each polymer. Combinations of GA and MD have been successfully applied to encapsulate agarwood oil, cardamom oil, procyanidins from grape seeds, and betalains from beetroot extract and produced encouraging results (Alves et al., 2014; Mkhari et al., 2023; Sahlan et al., 2019; Zhang et al., 2007). These studies demonstrated the potential of blending GA and MD to produce high‐quality capsules with properties desired by the food industry for functional food production.

Despite the growing interest in polymer blends among researchers, the use of GA and MD to encapsulate pomegranate seed oil has primarily focused on exploiting the individual characteristics of the polymers to produce capsules with desired qualities (Kalamara et al., 2015). However, no literature exists on the microencapsulation of PSO using a binary blend of GA and MD, which could potentially further augment the quality of the powders and provide the food industry with the much‐needed stable functional ingredients for functional food applications. To address this research gap, the current study aimed to evaluate the impact of the blending of GA and MD on the physicochemical, technofunctional, phytochemical, antioxidant, and morphological properties of freeze‐dried PSO powders and establish an optimum formulation for high‐quality PSO powders. The ultimate goal is to promote functional food development and a circular bioeconomy by creating stable, high‐quality PSO powders from pomegranate seed waste and maximizing the economic potential of pomegranates.

## 2 MATERIALS AND METHODS

### 2.1 Plant material and chemicals

Pomegranate fruit pomace (processing waste) from cv. Wonderful was procured from Ubali Pomegranate Farm in Pretoria, Gauteng Province, South Africa. Wall materials, including MD and GA, were procured from Sigma Aldrich, USA. All the other chemical reagents used in this study, including 2,4,6‐tri(2‐pyridyl)‐s‐triazine (TPTZ), Folin–Ciocalteu phenol reagent, gallic acid, and DPPH were of analytical grade and purchased from Sigma‐Aldrich, Johannesburg, South Africa.

### 2.2 Sample preparation and oil extraction

Pomegranate seeds were separated from the peels and membranes, thoroughly cleaned with tap water, and oven‐dried at 50°C for 24 h to achieve a moisture content of 12% (Kaseke et al., 2021a, 2021b). The dried seeds were stored at 4°C until further use. Prior to oil extraction, the seeds were ground to a uniform particle size of less than 1 mm. An ultrasound‐assisted solvent extraction technique was employed to extract pomegranate seed oil, as described by Kaseke et al. (2020a), (2020b). For this process, 200 g of pomegranate seed powder was mixed with 600 mL of 100% ethanol. The mixture was then sonicated at 40 ± 5°C for 40 min in an ultrasonic cleaner (Labotec, Sonic Clean). After sonication, the samples were filtered through a Whatman 1 filter paper, and the solvent was subsequently recovered using a BUCHI Rotavapor R‐300 (Postfach, CH‐9230).

### 2.3 Oil‐in‐water emulsion preparation, encapsulation and freeze‐drying

GA:MD blends (1:0, 0:1, 1:1, 3:1, and 1:3, respectively) were prepared according to the method described by Turasan et al. (2015) with slight modifications. The aim was to assess a number of economical combinations that would provide distinct results and recommend the optimum formulation (George et al., 2021; Mkhari et al., 2023). The blended wall materials (10%) were independently dissolved in 100 mL of distilled water with continuous stirring using a magnetic stirrer at 50°C and then homogenized using a Stuart SHM2 homogenizer (Staffordshire) for 1 min. The homogenized samples were left overnight for complete hydration, after which 15% PSO was independently added to each sample, and the mixture was further homogenized to form an emulsion. The emulsions were frozen at −80°C for 24 h before freeze‐drying in a freeze dryer at −30°C and 0.67 mbar for 24 h. The freeze‐dried samples were pulverized, packed into brown bottles, and stored at room temperature (20 ± 2°C).

### 2.4 Physical and techno‐functional properties of PSO powders

#### 2.4.1 Encapsulation yield

The encapsulation yield (EY) of the powder was calculated as the weight of the core material and wall materials divided by the ratio of the powders that were produced after drying, as shown in equation 1:

(1) $$\mathrm{EY}\;\left(\%\right)=\frac{\text{Weight of encapsulated seed oil powder}\;\left(g\right)}{\text{Weight of core and wall materials}\;\left(g\right)}\times 100\;$$

#### 2.4.2 Oil encapsulation efficiency

Oil encapsulation efficiency (EE) was determined according to Haq and Chun (2018). One gram of PSO powder was mixed with 30 mL of ethanol, and the mixture was vortexed for 30 s to extract the surface oil. The solvent was filtered twice before the residue was dried overnight in a fume hood and then oven‐dried at 105°C for 30 min. The difference in weight between the PSO powder and the residue was used to calculate surface oil. The encapsulation efficiency was then calculated using equation 2:

(2) $$\mathrm{EE}\;\left(\%\right)=\frac{\text{Total oil}-\text{surface oil}\;}{\;\text{total oil}\;}\times 100$$

where total oil is 15% PSO.

#### 2.4.3 Color and moisture content (MC)

Color attributes, including lightness (L*), redness (a*), and yellowness (b*) were measured using a calibrated chromometer (CR‐10 plus, Konica Minolta, Osaka, Japan). Hue angle (h°), Chroma (C*), and total color differences (∆E) were calculated using equations ((3), (4), (5)):

(3) $$h^{{}^{\circ}}–\tan^{-1}\;\left(\frac{b*}{a*}\right)$$

(4) $$C^{*}–\sqrt{a^{*2}+b^{*2}}$$

(5) $$\text{∆E}–{\left({\text{∆L}}^{*2}+{\text{∆a}}^{*2}+{\text{∆b}}^{*2}\right)}^{1/2}$$

Moisture content was analyzed using a moisture analyzer (KERN DBS60‐3 Balingen) at 100°C.

#### 2.4.4 Hygroscopicity, solubility, wettability, and bulk density

Hygroscopicity was measured by spreading 0.5 g of each powder evenly on Petri dishes (9 cm diameter) and storing them in a desiccator with a saturated sodium chloride solution (75% relative humidity) at 25°C for 7 days (Kori et al., 2022). The hygroscopicity was calculated by dividing the weight of the wet powder (after 7 days) by the weight of the dry powder (on day 0), with the result expressed as a percentage.

Solubility was assessed by dispersing 1 g of the powder in 50 mL of distilled water at room temperature (25 ± 2°C). The samples were mixed using a magnetic stirrer at 29 × g for 5 min, followed by centrifugation at 724 × *g* for 5 min. The supernatant (20 mL) was transferred to Petri dishes and oven‐dried at 70°C for 24 h. The powder solubility was calculated by dividing the weight of the dried supernatant by the initial weight of the powder (Kori et al., 2022).

To measure wettability, 1 g of powder was spread in a beaker containing 25 mL of distilled water. The time taken for all the powder particles to be completely immersed in the water was recorded and reported as wettability (Kori et al., 2022).

Bulk density was determined by adding 1 g of powder into a 25 mL calibrated measuring cylinder and measuring the height of the powder. The measuring cylinder was then gently tapped 20 times to compact the powder. The bulk density (g/cm^3^) was calculated by dividing the mass of the powder by its volume (Kori et al., 2022).

### 2.5 Microscopic surface morphology

The microscopic surface morphology of the powder particles was examined using a TESCAN Vega 3 scanning electron microscope (Brno). A thin layer of gold was applied to the powder samples using a sputter coater to enhance conductivity. The coated samples were then viewed under the scanning electron microscope (SEM) at magnifications of 100×. The resulting images were processed and analyzed using ImageJ software from the National Institutes of Health (Bethesda).

### 2.6 FT‐IR spectroscopy

To identify the functional groups present in the PSO fatty acids, FT‐IR spectroscopy was performed. A pellet containing PSO powder was prepared using the potassium bromide (KBr) method. The attenuated total reflection (ATR) method was employed to observe the absorption peaks in the powder. The FT‐IR spectrum was recorded using a Series 100 FT‐IR 1650 spectrophotometer (PerkinElmer), configured to operate in the 400–4000 cm^−1^ range.

### 2.7 Gas chromatography mass spectrometry (GC–MS/MS) analysis

The fatty acid methyl esters (FAMEs) were prepared following the method described by Mphahlele et al. (2017). The FAMEs were separated and analyzed using a GC–MS system (6890 N, Agilent Technologies Network) coupled to an Agilent Technologies Inert XL EI/CI Mass Selective Detector (MSD) (5975B, Agilent Technologies Inc). Helium served as the carrier gas at a flow rate of 0.017 mL/s. One microliter of the sample was injected into the system with a split ratio of 10:1. The oven temperature was initially set at 100°C, then increased to 180°C at a rate of 25°C/min and held for 3 min, further raised to 200°C at a rate of 4°C/min and held for 5 min, then increased to 280°C at a rate of 8°C/min, and finally to 310°C at a rate of 10°C/min, where it was held for 5 min. The profiles of the pomegranate seed oil fatty acids were identified by comparing their retention times with those of known standards. The relative content (%) of each fatty acid was calculated by dividing the peak area of each fatty acid by the total peak area of all identified fatty acids (Kaseke et al., 2021a, 2021b).

### 2.8 Total phenolic content and antioxidant activity

#### 2.8.1 Preparation of PSO powder extract

To prepare the PSO powder extract, 0.5 g of the encapsulated powder was mixed with 2.5 mL of 50% methanol. The mixture was vortexed for 30 s and then sonicated in an ultrasonic cleaner (Labotec, Sonic Clean) for 15 min. Following sonication, the samples were centrifuged (Thermo Scientific, Biofuge, Stratos) for 5 min at 8050 × g. The resulting supernatant was collected and used to determine total phenolic content and antioxidant activity.

#### 2.8.2 Total phenolic content (TPC)

The Folin–Ciocalteu (FC) method was used to determine the TPC of the PSO powder extracts (Fawole & Opara, 2013). The procedure involved mixing 50 μL of PSO powder extract with 450 μL of 50% methanol and 500 μL of FC reagent. This mixture was vortexed for 30 s and incubated in the dark for 10 min. After incubation, 2% sodium carbonate was added to the mixture, which was then incubated for an additional 40 min in the dark. Absorbance was measured at 725 nm using a SP‐UV 300 UV–Vis spectrophotometer, with 50% methanol serving as the blank. A standard curve was prepared using gallic acid (0–10 mg/mL; *R* ^2^ = .9889), and the results were expressed as mg gallic acid equivalent per g of PSO powder.

#### 2.8.3 Ferric‐reducing antioxidant power (FRAP)

The FRAP of the PSO powder extracts was determined using the method described by Fawole and Opara (2013). Briefly, 150 μL of PSO powder extract was mixed with 2.85 mL of FRAP reagent solution. The mixture was then incubated in the dark for 30 min. Absorbance was measured at 593 nm using a UV–Vis spectrophotometer (SP‐UV 300). A standard curve was prepared using Trolox (0–10 mM; *R* ^2^ = .9990), and the results were expressed as mM Trolox equivalents per g dry matter (mM TE/g DM).

#### 2.8.4 Radical scavenging ability (RSA)

The RSA of the PSO powder was measured using the 2.2‐diphenyl‐1‐picryl hydrazyl (DPPH) assay. The PSO powder extracts (15 μL) were mixed with 100% methanol (735 μL) and DPPH solution (750 μL). The samples were incubated at room temperature in darkness for 30 min, after which the absorbances were measured using a UV Visible Spectrophotometer (SP‐UV 300) at 517 nm. The RSA was calculated as a percentage according to equation (6).

(6) $$\mathrm{RSA}\;\left(\%\right)=\left(1-A/\text{Blank}\right)$$

where A = absorbance of RBJP extracts, and Blank = absorbance of DPPH solution.

### 2.9 Statistical analysis

Data were analyzed using one‐way analysis of variance (ANOVA) with the Statistica software v13 (TIBC) and SAS software (SAS Enterprise Guide 7.1; SAS Institute). Duncan's multiple‐range test at a 5% significance level was employed to separate the means, and results are presented as mean ± standard error (SE). Microsoft Excel version 16.0.13029.20344 (Microsoft Corporation) was used for graphical illustrations. The relationship between powder samples and quality attributes was established by performing a principal component analysis (PCA) using Microsoft Excel software with the XLSTAT 2019.4.1.63305 add‐in (Addinsoft).

## 3 RESULTS AND DISCUSSION

### 3.1 Encapsulation yield and efficiency

The results from Table 1 suggest that blending GA and MD at the studied ratios did not significantly (*p* > .05) affect the PSO EY. The EY ranged between 11.35% and 15.90%, which is lower than the EY reported in some literature. For instance, Comunian et al. (2020) reported more than double the EY when they produced spray‐dried PSO using GA as the wall material. The lower EY in the current study could have been influenced by the loss of PSO powder during the freeze‐drying process. Despite the lower EY, the EE, which demonstrates the actual amount of oil encapsulated within the biopolymer matrix, varied from 79.78% to 91.11% without significant variation (*p* > .05) across the GA:MD ratios. This suggests that EE was largely independent of the wall materials' blending ratios. Notably, the EE results were higher than those reported in previous studies that applied GA and MD as encapsulants. This indicates that the powders developed in this study had lower surface oil and that the biopolymers were efficient in encapsulating the PSO. In comparison, Calvo et al. (2011) and Quispe‐Condori et al. (2011) reported EE values ranging from 37% to 69% and 33% to 92%, respectively, for freeze‐dried GA and MD‐encapsulated walnut and flaxseed oils. Additionally, Özbek and Ergönül (2020) reported EE ranging from 62% to 71% for freeze‐dried pumpkin seed oil encapsulated with GA and MD. These observations suggest that a sufficient amount of PSO can be encapsulated in GA and MD composites for further use in formulating functional foods. The variation in EY and EE observed between the current study and the literature highlights the impact of the type of fixed oil, the concentration of the biopolymers, and the drying process on the formation of microcapsules (Concha et al., 2022). The relatively high EE observed in the present study may be attributed to the effective interaction between the GA and MD, which likely formed a strong matrix capable of efficiently entrapping the PSO. This efficiency in encapsulation is critical for the development of functional food ingredients, as it ensures that the bioactive components of the oil are well preserved and protected from environmental factors.

**TABLE 1:** Encapsulation yield (EY, %), encapsulation efficiency (EE, %), color attributes and moisture content (MC, %) of pomegranate seed oil powders obtained by blending GA and MD at different ratios.

	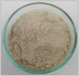	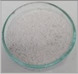	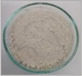	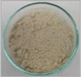	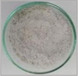
	GA:MD (1:0)	GA:MD (0:1)	GA:MD (1:1)	GA:MD (3:1)	GA:MD (1:3)
EY	12.78 ± 0.07^ab^	11.35 ± 0.26^b^	13.40 ± 0.60^ab^	13.01 ± 1.51^ab^	15.90 ± 1.51^a^
EE	91.11 ± 2.32^a^	90.67 ± 4.16^a^	85.33 ± 5.55^a^	79.78 ± 8.56^a^	81.78 ± 7.15^a^
MC	5.73 ± 0.44^b***^	7.32 ± 0.25^a^	4.59 ± 0.31^c^	6.77 ± 0.23^a^	7.04 ± 0.14^a^
L*	89.04 ± 1.25^b^	93.07 ± 0.97^a^	92.76 ± 0.29^a^	85.36 ± 1.27^c^	95 ± 0.46^a^
a*	−0.8 ± 0.30^b^	0.70 ± 0.41^a^	−1.29 ± 0.21^b^	−1.52 ± 0.3^b^	−0.78 ± 0.12^b^
b*	17.92 ± 0.64^a^	4.94 ± 0.16^d^	14.62 ± 0.28^b^	15.08 ± 0.60^b^	8.42 ± 0.11^c^
h°	−87.38 ± 1.03^b^	81.95 ± 4.69^a^	−84.92 ± 0.88^b^	−84.29 ± 1.09^b^	−84.73 ± 0.72^b^
C*	17.95 ± 0.63^a^	5.02 ± 0.15^d^	14.68 ± 0.26^b^	15.16 ± 0.62^b^	8.46 ± 0.12^c^
∆E	90.84 ± 1.11^b^	93.21 ± 0.96^ab^	93.92 ± 0.24^a^	86.71 ± 1.18^c^	95.37 ± 0.45^a^

*Note*: Values represent the mean ± SE of each assay in triplicates.

Abbreviations: ∆E, total color difference; a*, redness/greenness; b*, yellowness/blueness; C*, chroma; GA, gum Arabic; h°, hue angle; L*, lightness/darkness; MC, moisture content; MD, maltodextrin; WM, wall material.

***Each column with values assigned with different superscript letters is significantly different (*p* < .05) according to Duncan's multiple range tests.

### 3.2 Color and moisture content

The color attributes (L*, a*, b*, h°, and ΔE) of the PSO powders, along with the corresponding images, are presented in Table 1. PSO powders produced using MD alone, as well as those with GA and MD blended at 1:1 and 1:3, exhibited the highest L* values, indicating greater lightness. In contrast, powders produced using GA alone or with a GA:MD at 1:3 appeared more brownish, supporting their lower L* values. These results suggest that the inherent color of the biopolymers significantly impacted the lightness of the resulting powders. Overall, the PSO powders exhibited negative a* values, indicating a green hue and implying that the encapsulation process and biopolymers effectively masked any red pigments or anthocyanins present in the PSO. This finding aligns with the results reported by Ogrodowska et al. (2017), who observed negative a* values in pumpkin seed oil encapsulated with MD, whey protein isolate, and guar gum composites. Blending GA with MD significantly improved the b* (yellowness) values of the powders, particularly those produced using MD alone. This suggests that GA had a substantial influence on enhancing the yellowness of the powders. The highest b* value was observed in the GA:MD (1:0) formulation, indicating that GA either protected the PSO carotenoids effectively or had a minimal masking effect on the oil's natural color. Increasing the concentration of GA relative to MD also produced powders with a relatively better yellow hue compared to other GA:MD combinations.

The b* values observed in this study (ranging from 4.94 to 17.92) were comparable to those reported by Ogrodowska et al. (2017) for pumpkin seed oil powders produced from blends of maltodextrin, whey protein isolates, and guar gum (ranging from 9.5 to 24). All powders, except those produced using MD alone, exhibited negative h° values, indicating a more yellowish perception. This observation is consistent with the chroma (C*) results; powders produced with MD alone (5.02) or with a high MD concentration relative to GA (8.46) showed the lowest C* values, confirming that MD tended to mask the yellow color of the PSO. The highest ΔE values (93.21–95.37) were observed in PSO powders developed with GA:MD at 0:1, 1:3, and 1:1. These findings indicate significant color differences among these formulations, with MD driving the change.

Regarding MC, the PSO powders ranged from 4.59% to 7.32%. The highest MC was observed in the GA:MD (0:1) formulation, while the lowest was in the GA:MD (1:1) formulation. Lower moisture content is desirable as it contributes to the stability and shelf life of the powders. The variations in MC across different formulations highlight the influence of wall material composition on the moisture‐retention capacity of the powders.

Blending GA and MD at a ratio of 1:1 significantly reduced the MC of the PSO powder, making it 1.2‐ to 1.6‐fold lower compared to other formulations (Table 1). Specifically, the GA:MD (1:1) powder had an MC of 4.59%, suggesting that it could be more stable during storage under appropriate conditions compared to the other powders, which had MC values ranging from 5.73% to 7.32%. Increasing the concentration of GA significantly (*p* < .05) increased the MC, which can be attributed to its ramified structure and hydrophilic groups that enhance its capacity to bind water molecules from the environment (Nthimole et al., 2022). Conversely, increasing the concentration of MD in the binary blends had no significant impact on the powder's MC (*p* > .05; Table 1). According to the literature, an MC of less than 10% is desirable for all food powders to minimize the growth of spoilage microorganisms and biochemical reactions (Mercer & Peng, 2008). Therefore, all the PSO powders in this study exhibited this desired MC, which is crucial for the stability of powders during storage and their application in the food industry. Improved stability of food powders with less than 10% MC during storage has been reported in previous studies (da Silva et al., 2013).

### 3.3 Hygroscopicity, solubility, wettability, and bulk density

**FIGURE 1:**
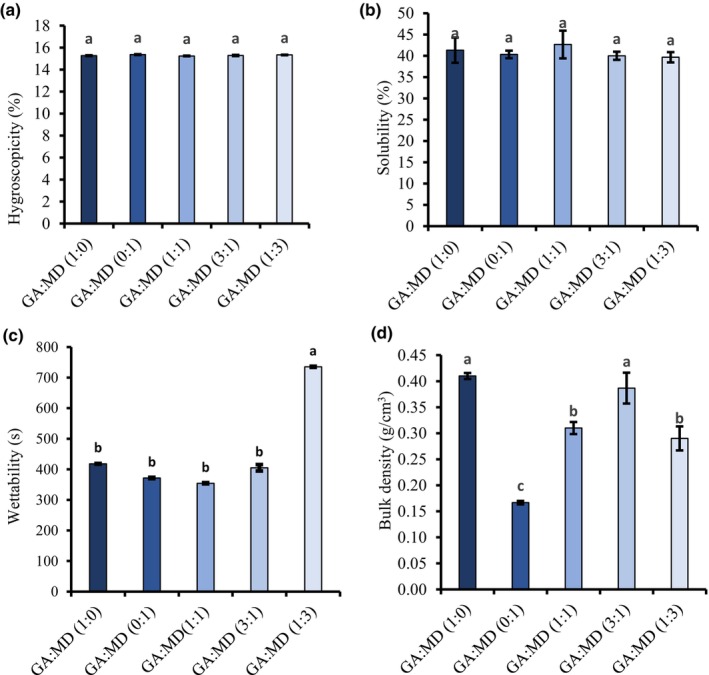
Chemical and technofunctional properties of freeze‐dried PSO powders encapsulated using GA and MD. Hygroscopicity (a), Solubility (b), Wettability (c), and Bulk density (d). Different letters on each bar show significant differences in the means (*p* < .05). Vertical bars indicate the standard deviation of the mean. GA, gum Arabic; MD, maltodextrin.

According to GEA NIRO (2005), powder hygroscopicity can be categorized as hygroscopic (15.1–20%), mildly hygroscopic (10%–15%), or non‐hygroscopic (≤10%). As depicted in Figure 1a, the hygroscopicity of the PSO powders ranged between 15.24% and 15.37%, indicating that all the powder samples are hygroscopic. Therefore, the PSO powders require proper packaging and storage conditions. No significant differences (*p* > .05) were observed in the powders' hygroscopicity, suggesting that blending GA and MD did not influence the powders' ability to absorb water from the environment. Contrasting results were reported in the literature. For example, Fernandes et al. (2013) reported that increasing the concentration of GA in rosemary essential oil powder significantly decreased hygroscopicity despite GA being a hygroscopic biopolymer. Karrar et al. (2021) reported hygroscopic values ranging from 6.95% to 8.76% for gurum seed oil powder produced using combinations of GA, MD, and whey protein isolate. These values are approximately half those observed in the present study. Factors such as powder particle size, MC, and the concentration of biopolymers could explain the observed differences and should be considered when interpreting hygroscopicity results from powders.

The PSO powders produced in this study showed poor solubility in water, ranging from 39.67% to 42.67%, likely due to the hydrophobic nature of lipids, which are immiscible in water (Figure 1b). There was no significant difference (*p* > .05) in solubility among the powder samples. These results are consistent with the findings of de Barros Fernandes et al. (2014), who reported solubility ranging from 41.85% to 47.72% for rosemary oil encapsulated using GA, MD, inulin, and modified starch through spray‐drying. Contrary to these results, Akram et al. (2021) reported twice as high solubility when encapsulating fish oil and flaxseed oil using GA and MD. Similarly, Kha et al. (2014) observed solubility of over 85% for spray‐dried gac oil using a mixture of GA and whey protein. The concentration of the oil and the particle size of the powder could account for the variation between the current solubility results and those reported in the literature.

Powder wettability, indicating the capacity of the powder to be reconstituted into other products, varied significantly (*p* < .05) from 354 s to 735 s (Figure 1c). The highest wettability time was observed in the GA:MD (1:3) powder, which could be attributed to its higher MC. Conversely, powders with a higher concentration of GA had lower wettability times, likely due to GA's hydrophilic properties that facilitated the diffusion of water particles, reducing the powders' wettability time (Bae & Lee, 2008). There is no consistent trend in the seed oil powder wettability results reported in the literature. For example, Fernandes et al. (2013) observed wettability times ranging from 155 to 481 s for GA‐encapsulated rosemary oil powder. In contrast, Akram et al. (2021) reported that flaxseed oil powder took 74.3 s (MD) and 83.2 s (GA) to become fully saturated. Karrar et al. (2021) observed significantly higher wettability times (765 s) for gurum seed oil encapsulated with a GA, MD, and whey protein isolate mixture. These varied results could reflect differences in powder particle sizes.

The bulk density of PSO powders in this study ranged from 0.17 g/cm^3^ to 0.41 g/cm^3^ (Figure 1d). Powders produced using GA alone had the highest bulk density (0.41 g/cm^3^), while the rest varied significantly (0.17–0.39 g/cm^3^). This suggests that GA:MD (1:0) and GA:MD (3:1) powders could be easier to package, transport, and store. Factors such as MC, particle size, particle‐size distribution, and particle shape can influence bulk density (Rosland Abel et al., 2020). The smaller particle sizes observed in the PSO powders produced using GA:MD (1:0) and GA:MD (3:1) may be linked to their higher bulk density. This also implies that these powders may exhibit lower lipid oxidation during storage, necessitating further studies on their stability (Premi & Sharma, 2017).

### 3.4 Morphology of encapsulated powders

**FIGURE 2:**
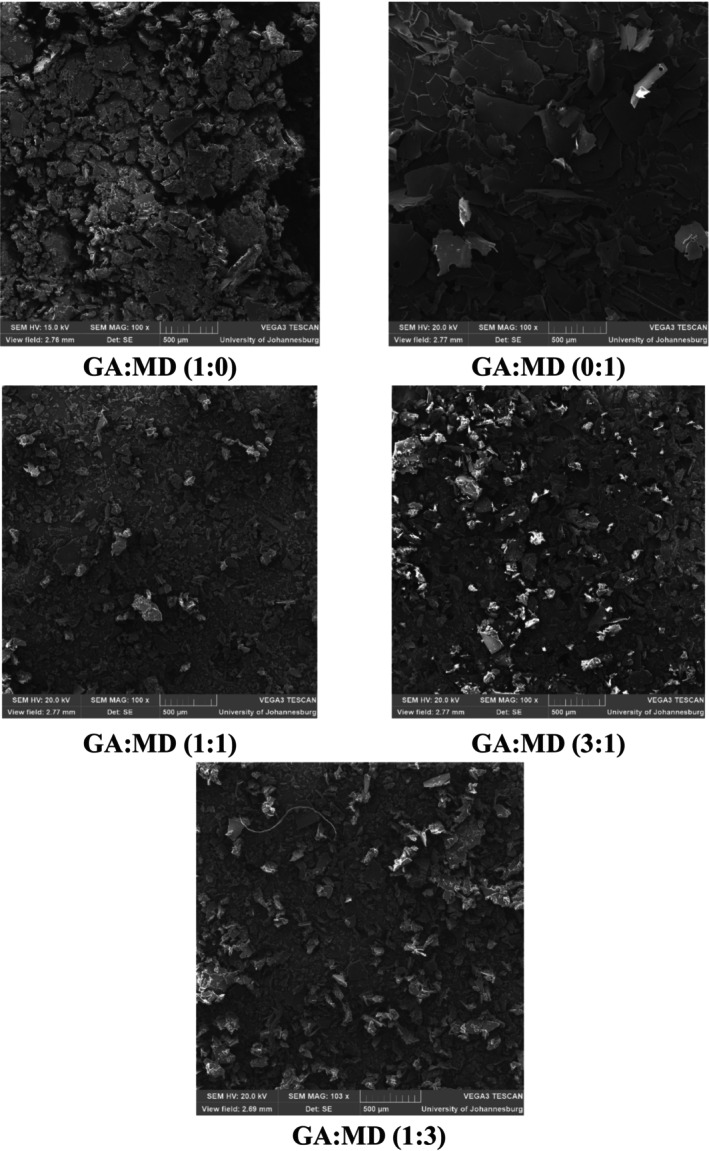
Scanning electron microscopy micrographs of encapsulated PSO using different combinations of gum Arabic (GA) and maltodextrin (MD) at 100x magnification.

The morphological structure of the PSO powder can provide insights into its stability during storage (Premi & Sharma, 2017). The SEM was used to examine and compare the microstructures of the freeze‐dried PSO powder samples, revealing significantly distinctive shapes and surface characteristics (Figure 2). The powders exhibited a range of particle morphologies, including flake‐like shapes, smooth surfaces, and wrinkled and porous large surface areas. These morphological features are consistent with those reported by Ogrodowska et al. (2017), who observed that GA and MD encapsulated pumpkin seed oil particles typically had flake‐like shapes with large, porous, and wrinkled surfaces. The presence of these characteristics is indicative of the complex structure formed during the freeze‐drying process. In addition, the freeze‐drying process promoted the development of crystals in blended wall materials, particularly when the concentration of GA was increased against MD. GA is known to prevent crystallization in food products (Maqbool et al., 2011), and therefore, the increased crystallization observed in this study when its concentration was trebled against MD might suggest structural modification of GA promoting crystal formation.

According to Nedovic et al. (2011), the freeze‐drying process forms a highly porous wall between the active agent and its surroundings, enhancing the protective capacity of the encapsulation matrix. Similarly, Özbek and Ergönül (2020) found that the surfaces of freeze‐dried GA and MD microcapsules of pumpkin seed oil were generally irregular, porous, and cracked, which they attributed to the effects of the grinding process. These structural attributes can influence the stability and release profile of the encapsulated oil. Anwar and Kunz (2011) also reported that freeze‐dried fish oil powders encapsulated with GA and MD displayed irregular, porous, and cracked surfaces, further supporting the findings observed in this study. The GA:MD (1:0) formulation displayed rough and irregular surfaces with numerous small and fragmented particles, attributed to GA's hydrophilic and emulsifying properties that lead to complex and highly ramified structures. This morphology enhances encapsulation and protection but produces a more complex particle structure.

In contrast, the GA:MD (0:1) formulation exhibited smoother surfaces with fewer and larger particles, reflecting MD's influence in forming more uniform and less complex structures. This simpler morphology may improve flow properties and ease handling but might offer less protection to the encapsulated oil compared to GA. The GA:MD (1:1) formulation showed a combination of rough and smooth surfaces with intermediate particle sizes, benefiting from the synergistic effects of GA and MD. This balanced morphology combines the protective features of GA with the uniformity provided by MD. For the GA:MD (3:1) formulation, the particles appeared rougher and more irregular than the GA:MD (1:1) blend but less so than the GA:MD (1:0) formulation, indicating that increasing GA concentration enhances structural complexity and protective capabilities while maintaining some uniformity. The GA:MD (1:3) formulation had a morphology similar to the GA:MD (0:1) blend, with smoother and more compact particles, suggesting that higher MD concentrations dominate the morphological characteristics, leading to a more uniform particle structure. These observations align with the physical and chemical properties discussed earlier. The rough and complex structures in GA‐dominant formulations (e.g., GA:MD 1:0 and 3:1) correlate with higher bulk density and potentially better EE due to the enhanced protective matrix. Conversely, the smoother and more uniform particles in MD‐dominant formulations (e.g., GA:MD 0:1 and 1:3) may facilitate better solubility and flow properties but might not offer the same level of protection as the encapsulated oil. To ascertain these phenomena, storage stability studies of the powders and correlation studies between the physical and chemical parameters of the powders are necessary.

### 3.5 FT‐IR spectroscopy of encapsulated PSO

FT‐IR spectroscopy is a powerful technique for identifying various functional groups within a sample (Khusro et al., 2014). This method relies on the physico‐chemical characteristics of the sample, enabling the detection of specific molecular vibrations that correspond to different functional groups (Cocchi et al., 2004). According to Purushothaman et al. (2016), the absorption intensity in FT‐IR produces a wide range of peaks at different frequencies, each indicative of particular functional groups.

**FIGURE 3:**
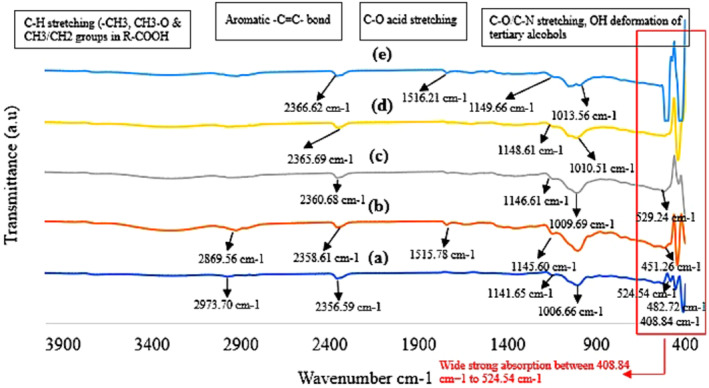
FT‐IR spectra for freeze‐dried pomegranate seed oil powder produced using different blends of gum Arabic (GA) and maltodextrin (MD). GA:MD (1:0) (a), GA:MD (3:1) (b), GA:MD (1:1) (c), GA:MD (1:3) (d), and GA:MD (0:1) (e).

The FT‐IR spectra of encapsulated PSO using different blending ratios of GA and MD (1:0, 0:1, 1:1, 3:1, and 1:3) were obtained using an FT‐IR spectrophotometer (FT/R‐4100 type A) over a spectrum range of 4000–400 cm^−1^ (Figure 3). The analysis revealed several key peaks, each corresponding to different functional groups in the encapsulated PSO. Between 3000 and 2000 cm^−1^, seven stretching bands were observed. Two prominent peaks at 2973.70 cm^−1^ and 2869.56 cm^−1^ indicated the presence of C–H aliphatic stretching, which is associated with methyl (−CH₃), methylene (>CH₂), methyne (>CH−), and special methyl (−CH₃) groups of the fatty acid chains in triacylglycerols (Coates, 2000; Rajiv et al., 2017; Zahed et al., 2024).

Additionally, five peaks at 2366.62 cm^−1^, 2365.69 cm^−1^, 2360.68 cm^−1^, 2358.61 cm^−1^, and 2356.59 cm^−1^ suggested the presence of aromatic bonds or N–H bending vibrations (Abdullah et al., 2020), indicating possible aromatic compounds within the oil. Alternatively, the N–H group could come from the GA, which is known to contain small amounts of proteins covalently bonded to the polysaccharide structure (Putro et al., 2023).

In the 2000–1500 cm^−1^ range, two significant peaks were observed at 1515.21 cm^−1^ and 1516.78 cm^−1^. These peaks indicate C–O stretching, which is typically associated with carboxylic acids and their derivatives. This suggests that the encapsulated oil retains significant amounts of these functional groups, which are crucial for maintaining the oil's chemical integrity and potential bioactivity. These observations concur with findings from previous studies (Jamshidi et al., 2020; Oliveira et al., 2016; Zahed et al., 2023). In addition, nine peaks were detected between 1500 and 600 cm^−1^, spanning from 1149.66 cm^−1^ to 1006.66 cm^−1^. These peaks are attributed to C–O stretching and –OH deformation, which are indicative of the presence of fatty acids and punicic acid (Kutlu et al., 2022). The presence of these peaks suggests that the encapsulation process has the potential to preserve punicic acid, which plays a role in the functional properties of the PSO. The variations in the FT‐IR spectra among different GA and MD blends highlight the impact of wall material composition on the chemical stability and functionality of the encapsulated PSO. For instance, the GA:MD (1:0) blend showed prominent C–H and C=O stretching vibrations, indicating effective preservation of aliphatic hydrocarbons and carboxylic acids, which are important components of fatty acids. Conversely, the GA:MD (0:1) blend displayed significant aromatic and hydroxyl group signals, reflecting MD's ability to stabilize these functional groups. The GA:MD (1:1) blend provided a balanced profile, combining features of both GA and MD, which suggests an optimal protective effect for the encapsulated oil. This balanced chemical environment might be beneficial for maintaining the bioactive compounds in PSO, thereby enhancing its functionality in potential applications.

### 3.6 Fatty acid composition

According to Table 2, the total saturated fatty acids (SFA) in the fresh PSO was 59.12 mg/g, which significantly decreased to between 4.35 and 12.18 mg/g after encapsulation, representing a 79%–93% decrease compared to fresh PSO. Comunian et al. (2020) established that spray‐drying encapsulation of PSO using whey protein isolate and its combination with modified starch did not affect the oil composition. The variation could be explained by differences in the pomegranate cultivar, origins of the oil, wall material, and encapsulation technique, among other factors. The GA:MD (0:1) and GA:MD (1:3) powders exhibited the lowest SFA content. Palmitic acid, the main SFA in PSO, decreased by 5‐ to 12‐fold after encapsulation. The GA:MD (1:0) powder exhibited the highest palmitic acid content at 6.80 mg/g, while the GA:MD (0:1) and GA:MD (1:3) powders exhibited the lowest at 2.95 mg/g. High levels of palmitic acid have been linked to inflammatory processes and metabolic disorders, so its reduction is desirable (Bojková et al., 2020). Stearic acid, which lowers low‐density lipoprotein cholesterol (LDL‐C) and reduces cancer risk (Hunter et al., 2010), showed the highest concentration in GA:MD (1:0) powder (3.78 mg/g) and the lowest in GA:MD (0:1) and GA:MD (1:3) powders. Other individual SFA, including lauric, myristic, pentadecyclic, arachidic, and behenic acids found in lower concentrations in PSO, also decreased after encapsulation (Table 2).

**TABLE 2:** Fatty acid composition and content (mg/g; %) in fresh and encapsulated pomegranate seed oil using different blends of GA and MD.

Fatty acid	PSO (mg/g:%)	GA:MD (1:0) (mg/g:%)	GA:MD (0:1) (mg/g:%)	GA:MD (1:1) (mg/g:%)	GA:MD (3:1) (mg/g:%)	GA:MD (1:3) (mg/g:%)
SFA						
Lauric acid (C12:0)	0.59 ± 0.00^a^ (0.11%)	0.47 ± 0.00^b^ (0.45%)***	0.40 ± 0.01^c^ (1.10%)	0.40 ± 0.01^c^ (0.46%)	0.48 ± 0.00^b^ (0.88%)	0.40 ± 0.01^c^ (1.10%)
Myristic acid (C14:0)	2.81 ± 0.00^a^ (0.51%)	0.98 ± 0.01^b^ (0.93%)	0.60 ± 0.00^c^ (1.64%)	0.92 ± 0.02^b^ (1.05%)	0.84 ± 0.02^b^ (1.54%)	0.60 ± 0.00^c^ (1.64%)
Pentadecyclic acid (C15:0)	0.24 ± 0.00^a^ (0.04%)	0.06 ± 0.00^b^ (0.06%)	0.04 ± 0.00^b^ (0.11%)	0.04 ± 0.00^b^ (0.05%)	0.04 ± 0.00^b^ (0.07%)	0.04 ± 0.00^b^ (0.11%)
Palmitic acid (C16:0)	35.30 ± 0.05^a^ (6.39%)	6.80 ± 0.01^b^ (6.47%)	2.95 ± 0.01^d^ (8.07%)	6.40 ± 0.01^b^ (7.33%)	4.08 ± 0.00^c^ (7.49%)	2.95 ± 0.01^d^ (8.07%)
Stearic acid (C18:0)	20.00 ± 0.02^a^ (3.62%)	3.78 ± 0.00^b^ (3.60%)	0.26 ± 0.00^d^ (0.71%)	2.69 ± 0.01^c^ (3.08%)	2.33 ± 0.00^c^ (4.28%)	0.26 ± 0.00^d^ (0.71%)
Arachidic acid (C20:0)	0.13 ± 0.00^a^ (0.02%)	0.07 ± 0.00^b^ (0.07%)	0.05 ± 0.00^c^ (0.14%)	0.05 ± 0.00^c^ (0.06%)	0.05 ± 0.00^c^ (0.09%)	0.05 ± 0.00^c^ (0.14%)
Behenic acid (C22:0)	0.05 ± 0.00^a^ (0.01%)	0.02 ± 0.00^b^ (0.02%)	0.05 ± 0.00^a^ (0.14%)	0.02 ± 0.00^b^ (0.02%)	0.03 ± 0.00^b^ (0.06%)	0.05 ± 0.00^a^ (0.14%)
**ΣSFA**	59.12 ± 0.07^a^ (10.69%)	12.18 ± 0.02^b^ (11.59%)	4.35 ± 0.02 ^a–d^ (11.91%)	10.52 ± 0.05 ^bc^ (12.04%)	7.85 ± 0.02 ^bc^ (14.40%)	4.35 ± 0.02^a–d^ (11.91%)
MUFA						
*trans‐*Oleic acid (C18:1 *trans*‐9)	0.04 ± 0.00^b^ (0.01%)	0.04 ± 0.00^b^ (0.04%)	0.05 ± 0.00^a^ (0.14%)	0.04 ± 0.00^b^ (0.05%)	0.03 ± 0.00^c^ (0.06%)	0.05 ± 0.00^a^ (0.14%)
*cis*‐Oleic acid (C18:1 *cis*‐9)	54.02 ± 0.09^a^ (9.77%)	10.75 ± 0.02^b^ (10.23%)	0.34 ± 0.00^e^ (0.93%)	0.64 ± 0.00^c^ (0.73%)	0.45 ± 0.00^d^ (0.83%)	0.34 ± 0.00^e^ (0.93%)
**ΣMUFA**	54.06 ± 0.09^ab^ (9.78%)	10.79 ± 0.02^b^ (10.27%)	0.39 ± 0.00^ae^ (1.07%)	0.68 ± 0.00^bc^ (0.78%)	0.48 ± 0.00^cd^ (0.88%)	0.39 ± 0.00^ae^ (1.07%)
PUFA						
Linoleic acid (C18:2)	93.48 ± 0.02^a^ (16.91%)	17.57 ± 0.02^b^ (16.73%)	6.50 ± 0.01^e^ (17.79%)	12.06 ± 0.01^c^ (13.81%)	9.58 ± 0.01^d^ (17.58%)	6.50 ± 0.01^e^ (17.79%)
γ‐Linolenic acid (C18:3)	2.48 ± 0.01^a^ (0.45%)	0.11 ± 0.00^b^ (0.11%)	0.04 ± 0.00^c^ (0.11%)	0.10 ± 0.00^b^ (0.11%)	0.05 ± 0.00^c^ (0.09%)	0.04 ± 0.00^c^ (0.11%)
α‐Linolenic acid (C18:3)	0.66 ± 0.00^a^ (0.12%)	0.49 ± 0.01^b^ (0.47%)	0.27 ± 0.00^d^ (0.74%)	0.49 ± 0.00^b^ (0.56%)	0.40 ± 0.00^c^ (0.73%)	0.27 ± 0.00^d^ (0.74%)
Punicic acid (C18:3)	343.00 ± 1.49^a^ (62.05%)	63.91 ± 0.02^b^ (60.84%)	24.99 ± 0.03^d^ (68.39%)	63.51 ± 0.25^b^ (72.70%)	36.14 ± 0.04^c^ (66.31%)	24.99 ± 0.03^d^ (68.39%)
**ΣPUFA**	439.62 ± 1.52^a^ (79.53%)	82.08 ± 0.05^b^ (78.13%)	31.80 ± 0.04^c–e^ (87.03%)	76.16 ± 0.26^bc^ (87.18%)	46.17 ± 0.05^cd^ (84.72%)	31.80 ± 0.04^c–e^ (87.03%)
ΣUFA	493.68 ± 1.61^ab^ (89.31%)	92.87 ± 0.07^b^ (88.4%)	32.19 ± 0.04^a–e^ (88.1%)	76.84 ± 0.26^bc^ (87.96%)	46.65 ± 0.05^cd^ (85.6%)	32.19 ± 0.04^a–e^ (88.1%)
ΣMUFA/ΣPUFA index	0.12 ± 0.06^a^ (0.02%)	0.13 ± 0.4^b^ (0.12%)	0.01 ± 0.00^c‐d^ (0.03%)	0.01 ± 0.00^bc^ (0.01%)	0.01 ± 0.00^cd^ (0.02%)	0.01 ± 0.00^cd^ (0.03%)
ΣUFA/ΣSFA index	8.35 ± 23^ab^ (1.51%)	7.62 ± 3.5^b^ (7.25%)	7.4 ± 2.0^a–e^ (20.25%)	7.3 ± 5.2^bc^ (8.36%)	5.94 ± 2.5^b–d^ (10.9%)	7.4 ± 2.0^a–e^ (20.25%)
ΣPUFA/ΣSFA index	7.44 ± 21.71^ab^ (1.35%)	6.74 ± 2.5^a–d^ (6.42%)	7.31 ± 2.00^b–e^ (20.01%)	7.24 ± 5.2^bc^ (8.29%)	5.88 ± 2.5^b–d^ (10.79%)	7.31 ± 2.00^b–e^ (20.01%)

Abbreviations: GA, gum Arabic; MD, maltodextrin; MUFA, *monounsaturated fatty acid*; PUFA, polyunsaturated fatty acid; SFA, saturated fatty acids; UFA, unsaturated fatty acids.

***Each column with values assigned with different superscript letters is significantly different (*p* < .05) according to Duncan's multiple range tests.

The total monounsaturated fatty acid (MUFA) content significantly decreased after encapsulation (Table 2). Unlike in the present study, the use of whey protein isolate combined with modified starch to encapsulate PSO insignificantly affected the MUFA composition (Comunian et al., 2020). In the current study, total MUFA in the encapsulated samples varied from 0.39 to 10.79 mg/g, with the GA:MD (1:0) powder exhibiting the highest MUFA content (10.79 mg/g). The main MUFAs identified were *trans*‐ and *cis*‐oleic acids, which are known for their protective effects against free radical damage, cardiovascular benefits, and anti‐inflammatory properties (Kwa et al., 2019). The GA:MD (1:0) powder exhibited the highest *cis*‐oleic acid content at 10.75 mg/g, while the other samples ranged between 0.34 and 0.64 mg/g. *Cis*‐oleic acid is beneficial for reducing cholesterol levels and lowering the risk of cardiovascular diseases (Oteng & Kersten, 2020).

The total polyunsaturated fatty acid (PUFA) content varied from 31.80 mg/g to 82.08 mg/g, representing 78%–87% of the total PSO fatty acids and were within the range reported by Yücetepe et al. (2021). PSO presents a composition that is rich in PUFA, which requires to be stabilized during storage. GA:MD (0:1) blend exhibited the highest PUFA concentration, while GA:MD (1:0) and GA:MD (1:3) showed the lowest. The main PUFA identified included linoleic acid (LA), γ‐linolenic acid (γ‐LA), α‐linolenic acid (ALA), and punicic acid (PA). Encapsulation significantly decreased their concentrations and could have been caused by either oxidation or losses during encapsulation. LA, crucial for cell membrane integrity (Mukerjee et al., 2021), was highest in the GA:MD (1:0) and GA:MD (1:1) powders at 17.57 mg/g and 12.06 mg/g, respectively. PA, the major fatty acid in PSO with numerous health benefits, showed a significant reduction after encapsulation, decreasing by 5–13 times. However, the GA:MD (1:0) and GA:MD (1:1) powders retained the highest levels of PA at 63.91 mg/g and 63.53 mg/g, respectively, which were 1.7–2.5‐fold higher than the other powders. The PUFA/SFA and UFA/SFA indices provide further insights into the fatty acid profiles. The PUFA/SFA index was highest in the GA:MD (0:1) formulation (7.62), indicating a higher proportion of PUFA than SFA. The UFA/SFA index was also highest in this formulation, reflecting the overall retention of unsaturated fatty acids (UFA). In the diet, PUFA can lower serum cholesterol and low‐density lipoprotein cholesterol levels, while SFA raises serum cholesterol levels. Consequently, a higher UFA/SFA ratio in GA:MD (0:1) formulation denotes a more positive effect toward the prevention of cardiovascular diseases (Kwa et al., 2019).

### 3.7 Total phenolic content, DPPH radical scavenging activity, and ferric‐reducing antioxidant power

**FIGURE 4:**
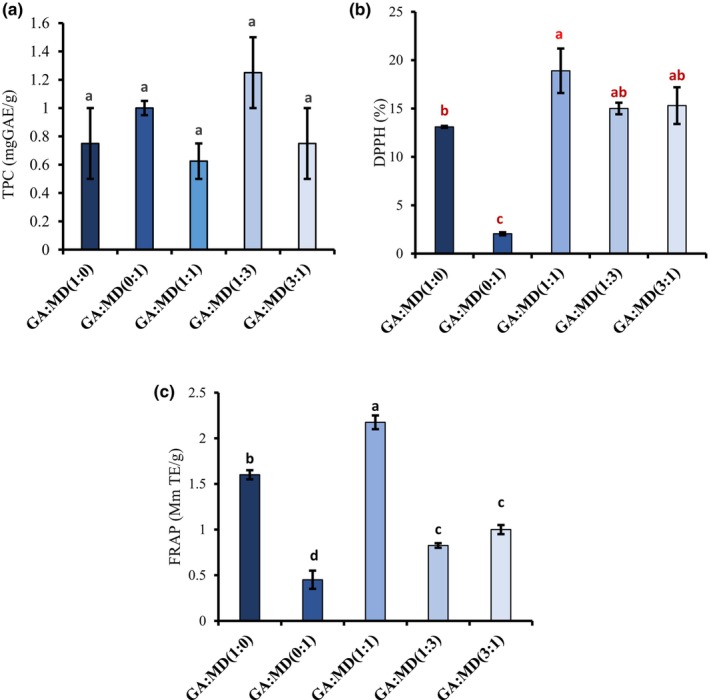
Phytochemicals and antioxidant activity of freeze‐dried PSO powders encapsulated using GA and MD blends. Total phenolic content (TPC) (a), DPPH radical scavenging activity (b), and ferric‐reducing antioxidant power (FRAP) (c). Different letters on each bar show significant differences in the means (*p* < .05). Vertical bars indicate the standard deviation of the mean. GA, gum Arabic; MD, maltodextrin.

Phenolic compounds have garnered significant attention among researchers due to their preventive effects on central neurodegenerative diseases, diabetes mellitus, cardiovascular diseases, and cancer (Özbek & Ergönül, 2020). While seed oils are not typically rich sources of phenolic compounds, protecting these compounds during processing and storage is crucial to maximizing their biological activities. As shown in Figure 4a, the total phenolic content (TPC) of the PSO powder samples ranged from 0.63 mg GAE/g to 1.25 mg GAE/g and did not significantly differ (*p* > .05) among the samples. These results are consistent with those reported by Özbek and Ergönül (2020), who found that TPC values did not vary significantly between GA‐ and MD‐encapsulated and freeze‐dried pumpkin seed oils. Despite the lack of significant differences, the GA:MD (0:1) and GA:MD (1:3) powders exhibited the highest TPC values, suggesting that MD had a more protective effect on the phenolic compounds in PSO during encapsulation. The literature highlights MD's ability to create a barrier or protective film that entraps phenolic compounds, preventing them from undergoing conformational changes due to external influences. However, Tolun et al. (2016) reported that the highest TPC was found in grape pomace extract powder, where GA and MD were mixed in equal proportions. Therefore, accurately interpreting the effect of biopolymers on preserving bioactive compounds from plant materials requires careful consideration of biopolymer concentration, plant material type, and encapsulation method.

Given the complexity of phytochemicals in plant extracts, no single assay can accurately reflect overall antioxidant activity. To assess the impact of blending GA and MD on the antioxidant activity of the developed PSO powders, the current study employed both DPPH and FRAP assays. Figure 4b,c illustrate the DPPH radical scavenging activity and FRAP for all powder formulations, respectively. The DPPH radical scavenging activity of the PSO powders, as shown in Figure 4b, ranged from 2.1% to 18.9%. The powders produced using GA and MD alone exhibited DPPH radical scavenging activities of 13.1% and 2.1%, respectively. Blending GA and MD improved the DPPH radical scavenging activity of the powders, increasing the activity to between 15% and 18.8%. Despite this improvement, the overall DPPH radical scavenging activity observed in this study was lower than expected. This could indicate the loss of some bioactive compounds responsible for the antioxidant activity of PSO, such as tocopherols, phytosterols, and carotenoids, during the encapsulation process. Interestingly, while the literature often commends MD for its protective effects on bioactive phytochemicals, the current study found that using MD alone to encapsulate PSO resulted in the lowest DPPH radical scavenging activity.

The FRAP assay, which measures the capacity of antioxidant compounds to convert Fe^3+^ to Fe^2+^ ions, showed a similar trend to the DPPH results but did not align with the TPC results (Figure 4c). This discrepancy suggests that TPC may not be the main bioactive compound contributing to the FRAP and DPPH radical scavenging activity of the encapsulated PSO. Other compounds, such as carotenoids, tocopherols, phytosterols, and fatty acids like punicic acid, should be considered to better understand the contributions of various PSO compounds to their antioxidant activity. The FRAP values for the PSO powders produced using GA and MD separately were 1.6 mM TE/g and 0.5 mM TE/g, respectively. Blending GA and MD at a 1:1 ratio significantly improved the FRAP of the developed powders (*p* < .05) compared to other blending ratios. Further increasing the concentrations of either GA or MD did not result in significant changes in FRAP values. The observation that encapsulating PSO with MD alone exhibited poor DPPH radical scavenging activity and FRAP highlights the need for further studies on the impact of biopolymers on the broad spectrum of antioxidant compounds in PSO and how they correlate with different antioxidant activity assays.

### 3.8 Principal component analysis

**FIGURE 5:**
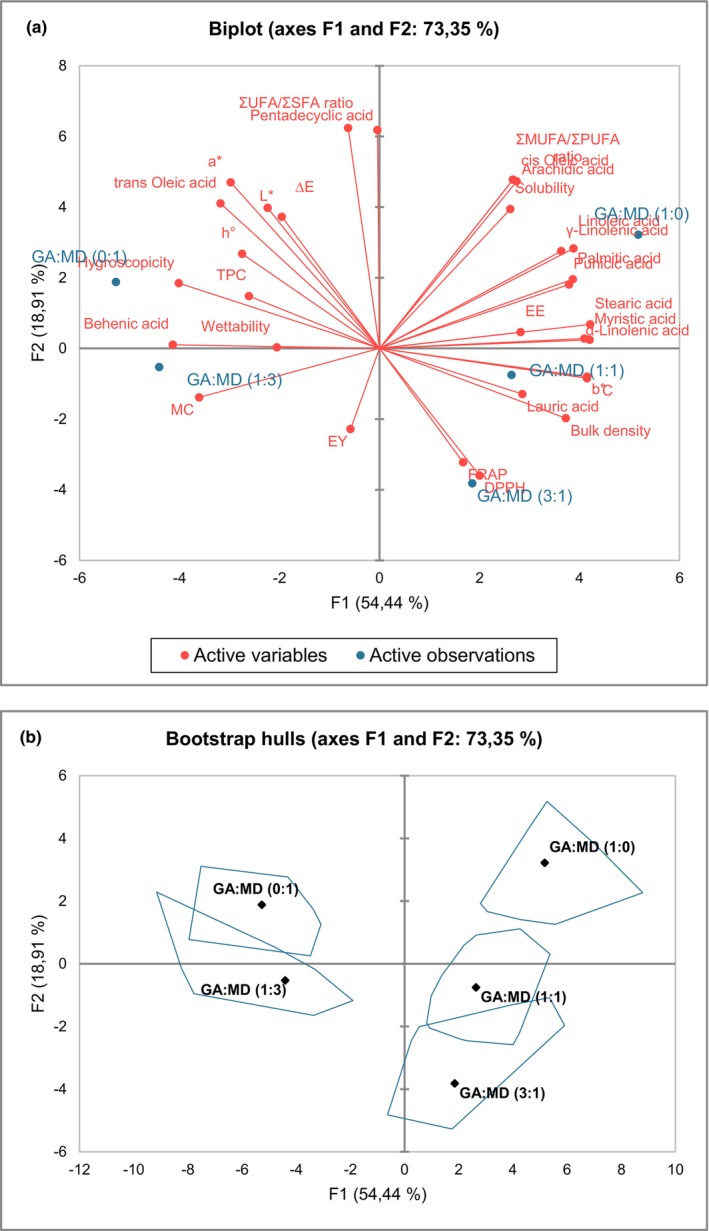
Principal component analysis (PCA) biplot (a) showing the relationship between the quality attributes (active variables) and the powder from the different gum Arabic (GA) and maltodextrin (MD) blending ratios (active observations) and PCA bootstrap hulls (b) showing confidence areas of the powders. ∆E, total color difference; a*, redness/greenness; b*, yellowness/blueness; C*, chroma; DPPH, 2,2‐ diphenyl‐1‐picrylhydrazyl; EE, encapsulation efficiency; EE, encapsulation yield; FRAP, ferric reducing antioxidant power; h°, hue angle; MC, moisture content; MUFA, *monounsaturated fatty acid*; PUFA, polyunsaturated fatty acid; SFA, saturated fatty acids; TPC, total phenolic content; UFA, unsaturated fatty acids.

The variability of the data generated in the present study was explained mainly by the first two principal components, F1 (54.44%) and F2 (18.91%), which together accounted for 73.35% of the total variability (Figure 5a). The analysis revealed significant differences in the properties of PSO powders based on the blending ratios of gum Arabic (GA) and maltodextrin (MD). The F1 plane primarily distinguished between GA:MD (1:0) and GA:MD (1:1) powders on the positive side and GA:MD (0:1) and GA:MD (1:3) powders on the negative side. The positive side of F1 was associated with better color attributes (b*, C*), higher bulk density, greater solubility, superior encapsulation efficiency (EE), and preservation of essential fatty acids such as myristic acid, palmitic acid, lauric acid, stearic acid, cis‐oleic acid, α‐linolenic acid, arachidic acid, γ‐linolenic acid, linoleic acid, and punicic acid. Additionally, the GA:MD (1:0) and GA:MD (1:1) powders exhibited favorable MUFA/PUFA ratios, indicating a balanced fatty acid profile that is beneficial for functional food applications. These findings suggest that these formulations provide better color and technofunctional properties while effectively preserving the essential fatty acids in PSO.

On the negative side of the F1 plane, GA:MD (0:1) and GA:MD (1:3) powders were correlated with higher moisture content (MC), a* (redness), hygroscopicity, trans‐oleic acid, and behenic acid. These characteristics suggest that these powders might be less stable during storage due to higher moisture uptake and less desirable fatty acid profiles. The F2 plane further differentiated the samples, with GA:MD (1:0) powders on the positive side associated with a*, pentadecyclic acid, arachidic acid, cis‐oleic acid, the MUFA/PUFA ratio, and the UFA/SFA ratio. This indicates that GA:MD (1:0) powders not only have better color properties but also maintain a favorable balance of unsaturated to saturated fatty acids, which is important for nutritional quality.

Conversely, GA:MD (3:1) powders were positioned on the negative side of the F2 plane and were strongly correlated with antioxidant properties, as indicated by FRAP and DPPH assays. This suggests that increasing the concentration of GA relative to MD enhances the antioxidant activity of the powders, making them suitable for applications where oxidative stability is critical. The agglomerative hierarchical clustering (Figure S1) and the bootstrap hulls plot (Figure 5b) further supported these findings by grouping GA:MD (1:0), GA:MD (1:1), and GA:MD (3:1) powders together, separating them from GA:MD (0:1) and GA:MD (1:3) powders. This clustering indicates that GA‐dominant formulations share similar desirable qualities, while MD‐dominant formulations form a distinct group with different properties.

Overall, the PCA and clustering analyses suggest that using GA alone, in equal concentration with MD, or in higher concentrations than MD produces PSO powders with desirable characteristics for the development of functional foods. These formulations offer improved color, stability, encapsulation efficiency, and preservation of essential fatty acids and antioxidant properties, making them suitable for various food applications.

## 4 CONCLUSIONS

This study encapsulated pomegranate seed oil (PSO) into powder form using gum Arabic (GA) and maltodextrin (MD) and their binary blends, offering an effective method to preserve the oil and expand its applications. The principal component analysis (PCA) indicated that while no single formulation achieved all the desired outcomes, certain blends provided distinct advantages. The GA:MD (1:0) and GA:MD (1:1) formulations produced powders with superior color, technofunctional properties, and better preservation of essential fatty acids compared to GA:MD (0:1) and GA:MD (1:3). These findings suggest that these specific ratios are particularly effective in maintaining the quality and stability of PSO during the encapsulation process.

Furthermore, the GA:MD (3:1) blend demonstrated the best antioxidant properties, as evidenced by higher FRAP and DPPH radical scavenging activity. This indicates that increasing the concentration of GA relative to MD can enhance the antioxidant capacity of the encapsulated PSO, making it a valuable formulation for applications where oxidative stability is critical.

These findings have significant practical implications for the food industry. The ability to encapsulate PSO using GA and MD blends provides a multipurpose approach to incorporating this specialty oil into functional foods, thereby promoting its health benefits and extending its shelf life. The study highlights the importance of selecting appropriate wall material ratios to optimize encapsulation. Using GA alone, in equal concentration with MD, or increasing its concentration to 3 fold of MD are recommended strategies for protecting the polyphenols and antioxidant activity of PSO, thereby producing powders with qualities desired by the food industry. These formulations promote a circular economy by utilizing by‐products like PSO effectively. Given the observed decrease in primary fatty acids after encapsulation, future research should focus on the stability of encapsulated PSO during storage, degradation kinetics, and its application in producing functional foods.

## AUTHOR CONTRIBUTIONS

**Kutloano Mangope:** Formal analysis (equal); investigation (equal); methodology (equal); writing – original draft (equal). **Tafadzwa Kaseke:** Supervision (equal); validation (equal); writing – review and editing (equal). **Olaniyi A. Fawole:** Conceptualization (equal); funding acquisition (equal); resources (equal); supervision (equal); writing – review and editing (equal).

## CONFLICT OF INTEREST STATEMENT

The authors declare that they have no conflict of interest.

## Supporting information


Figure S1.

## Data Availability

Data will be made available on request.
