# Supplementary material for: Microencapsulation and characterization of pomegranate seed oil using gum Arabic and maltodextrin blends for functional food applications
Source: Food Sci Nutr. 2024 Sep 30;12(11):9252–67. doi: 10.1002/fsn3.4493 (PMC11606825; doi:10.1002/fsn3.4493)
Supplement: Supplementary file 1 — Figure S1. [file FSN3-12-9252-s001.docx]

**Figure S1.** Agglomerative Hierarchical Clustering (AHC) dendrograms of the pomegranate seed oil powder from the different gum Arabic (GA) and maltodextrin (MD) blending ratios.
